# Patient-reported QoL in anal cancer survivors 3 and 6 years after treatment—results from the Swedish national ANCA study

**DOI:** 10.1007/s00520-021-06769-7

**Published:** 2022-01-26

**Authors:** Anna Axelsson, Mia Johansson, David Bock, Eva Haglind, Hanna de la Croix, Per J. Nilsson, Eva Angenete

**Affiliations:** 1 Department of Surgery, SSORG-Scandinavian Surgical Outcomes Research Group, Institute of Clinical Sciences, Sahlgrenska Academy, University of Gothenburg, Gothenburg, 416 85 Sweden; 2 Department of Oncology, Region Västra Götaland, Sahlgrenska University Hospital/Sahlgrenska, 413 45 Gothenburg, Sweden; 3 Department of Oncology, Institute of Clinical Sciences, Sahlgrenska Academy, University of Gothenburg, Gothenburg, Sweden; 4 Department of Surgery, Region Västra Götaland, Sahlgrenska University Hospital/Östra, Gothenburg, Sweden; 5 Department of Surgery, Div. of Coloproctology, Karolinska University Hospital, Stockholm, Sweden

**Keywords:** Anal cancer, Late side-effects, Survivorship, QoL, Bother of functional symptoms

## Abstract

**Purpose:**

The impact of anal cancer treatment for the patients is best evaluated by the patients themselves. The purpose of this study was to investigate quality of life (QoL) in patients with anal cancer at 3 and 6 years after treatment.

**Methods:**

A Swedish national cross-sectional prospective cohort study with patients diagnosed with anal cancer between 2011 and 2013. Patients were invited to respond to a QoL questionnaire at 3 and 6 years, with focus on bowel, urinary and sexual function, social and mental function, co-morbidity, lifestyle, daily activities, personal characteristics, and perceived QoL. It also contained questions on the severity of the symptoms regarding occurrence, frequency, and duration and the level of “bother” experienced related to functional symptoms.

QoL and prevalence of bother with urinary, sexual, bowel dysfunction, and anal pain were described. The prevalence of impaired QoL was compared with a healthy reference population. The association between QoL and experiencing bother was quantified by regression models.

**Results:**

From an original cohort of 464 patients with anal cancer, 264 (57%) were alive and contacted at 3 years and 230 (50%) at 6 years. One hundred ninety-five (74%) patients responded to the 3-year and 152 (66%) to the 6-year questionnaire. Sixty percent reported low QoL at both 3 and 6 years. Impaired QoL was more prevalent among patients with major bother due to bowel dysfunction (at 3 years RR 1.42, 95% CI (1.06–1.9) *p*-value 0.020, at 6 years RR 1.52, 95% CI (1.03–2.24) *p*-value 0.034) and urinary dysfunction (at 6 years RR 1.44, 95% CI (1.08–1.91) *p*-value 0.013). There was a tendency to a positive relationship between the number of bodily functions causing bother and risk for impaired QoL.

**Conclusion:**

Patients treated for anal cancer reported bother regarding several bodily functions as well as poor QoL both at 3 and 6 years without much improvement. Bother was also associated with low QoL indicating that function-related bother should be addressed.

## Introduction

Treatment for anal cancer has improved immensely over the past decades with combined chemoradiation now being the primary therapeutic option and surgery mainly reserved for patients with incomplete response or recurrence [8, 15]. In recent trials of different treatment regimens, 5-year survival rates of approximately 80% are reported [13]. The high curation rates lead to a sizeable cohort of anal cancer survivors and, thus, the concept of survivorship, i.e., living with long-term side effects related to the received treatment [1, 19] becomes increasingly important.

Radiotherapy causes structural changes in exposed organs i.e., bowel, anal sphincter, genital organs, and bladder. It may affect bowel, urinary and sexual function and can also lead to impaired musculoskeletal function and mobility [6, 14, 24, 28]. The radiation dose and the irradiated volume are important factors influencing adverse late side effects but individual factors such as genetic susceptibility and smoking are also believed to be of importance [1, 16]. Symptoms and impairment of function may have an impact on the patient’s quality of life (QoL), but to what extent can only fully be estimated through the patient’s own assessment [1, 19]. As side effects caused by radiotherapy can be progressive and develop years after exposure long-term follow-up is of importance, or the burden of symptoms experienced by anal cancer survivors may be underestimated [19]. Previous studies on QoL in patients with anal cancer and long-term follow-up were presented in a review performed by Sodergren et al. describing 11 studies using mainly EORTC QoL questionnaire (EORTC QLQC-30 or EORTC QLQCR-29). Results from these studies showed that bowel problems with diarrhea and impaired sexual function were the most common areas affecting QoL, and conclusion from the review was that there was a need for QoL questionnaire more specific for patients with anal cancer in future studies [19]. This has now been put into place, and there is a new EORTC questionnaire for patients treated for anal cancer (EORTC-ANL27) [18].

Our hypothesis was that there was a deterioration in QoL between 3 and 6 years of follow-up indicating a need for a long-term follow-up. We also hypothesized that low QoL correlated to one or more impaired body functions, as has been seen in previously in patients treated for rectal and prostate cancer [26, 27]. The aim of this study was to describe patient-reported QoL and bother due to dysfunction in bodily functions in patients treated for anal cancer at 3 and 6 years after conclusion of treatment and to study the relationship between QoL and bother [19].

## Material and methods

### Study design

The ANal CAncer study (ANCA) is a national cross-sectional study regarding QoL and functional outcome in patients with anal cancer. The study is based on a Swedish national cohort of patients diagnosed with anal cancer between January 2011 and December 2013 identified through the Swedish Cancer Register at the Swedish National Board of Health and Welfare.

### Data collection

Following patient approval, clinical data was collected from the Patient register at the Swedish National Board of Health and Welfare and from patient charts, collected from Swedish hospitals, using a standardized procedure with pre-specified clinical record form (CRF). Only patients with invasive squamous cell carcinoma of the anus were included in the study and invited to respond to a study-specific questionnaire at 3 and 6 years after diagnosis. The questionnaire was constructed according to a well-established method described elsewhere [21]. At the time when the study was designed, there was no pre-existing anal specific QoL instrument in use and for example, EORTC QLQ-C30 was considered to be too unspecific to answer our research question, and therefore, the decision was made to construct a more suitable questionnaire. The questionnaire included in total 260 questions with focus on bowel, urinary and sexual function, social and mental function, co-morbidity, lifestyle, daily activities, personal characteristics, and perceived QoL. It also contained questions about the severity of symptoms of occurrence, frequency and duration, and about the level of bother experienced relating to function. The 29-item Sense of Coherence scale (SOC-29) was also included [3]. Not all questions and instruments were used in the analysis for this sub-study, since focus was on QoL, explanatory variables, and bother regarding functional outcomes.

### Administration of questionnaires

Initially, all patients in the cohort received a letter with information concerning the study and an invitation to participate. A few days later, the patients were contacted by telephone from a research nurse to obtain consent to study inclusion and permission to send out the questionnaire. Two weeks after the questionnaire was sent, the patients received a postcard with a thank you note and a reminder if the questionnaire had not been returned. One final reminder by telephone was attempted after the initial contact. Previously, this procedure has achieved an overall response rate of approximately 90% [2, 5, 10].

### Outcome measures and possible explanatory variables

The primary endpoint was patient-reported QoL at 3 and 6 years. This was assessed in the question “how would you describe your QoL in the past month?” The response categories were presented in a Likert scale from 0 to 6 with 0 = no QoL and 6 = the best possible QoL. The response options were then dichotomized as has been done previously (0–4 = low QoL and 5–6 = good QoL) [21].

Potential explanatory variables for low QoL were selected both through clinical expertise and previously published results. Sense of coherence has previously been reported to impact on QoL in patients with rectal cancer and was therefore included [4]. Depression may affect QoL and was explored using the question: “would you call yourself depressed?” with response categories no, yes, or don’t know. This question has previously been validated in relation to the Hospital Anxiety Depression Scale [17]. Functional impairment (bowel, urinary, sexual) and anal pain were all considered potential explanatory variables. Bother regarding functional impairments was evaluated using questions about the level of bother from symptoms. For example: “how would you feel if this last month’s bowel impairment was to remain the same for the rest of your life?”. Each bother question had five response categories: “Not relevant, I haven’t had any bowel impairment the last month,” “It wouldn’t bother me at all,” “It would bother me slightly,” “It would bother me moderately,” and “It would bother me very much.”

The responses were grouped into three categories: no bother (no problems thus no bother), minor bother (no or slight bother), and major bother (moderate or much bother). Other factors included as potential explaining variables were self-reported comorbidity, socio-economic status, and type of treatment for anal cancer.

### Reference population

For comparison, we used a reference population of 1078 Swedish persons who answered identical questions included in a questionnaire described elsewhere [7]. The reference cohort was randomly selected from the Swedish population through the Swedish Tax Agency and completed the questionnaire between 2014 and 2015. The reference population was born between 1924 and 1983 with a median age of 63 years on accrual and a female:male ratio of 53%:47% [7].

### Statistical analysis

The prevalence of low QoL in the study population was assessed by a generalized linear multi-level model with a logit link and Bernoulli distribution [23]. A random intercept was used to account for the intra-patient dependence of the longitudinal data. Gender and time were included as a fixed effect and age as a continuous covariate as well as two- and three-way interaction effects in order to allow for synergy effects. Individual random effect (conditional) predictions as well as least-square mean fixed effect (marginal) predictions with 95% confidence intervals (CI) were displayed graphically. The mean effects of age were evaluated at the first and third quantiles of age at inclusion, 58 and 71 years, respectively. The prevalence in the reference population was reported using crude rates with 95% CI. The relationship between bother and the prevalence of low QoL was evaluated by regression analysis using the modified Poisson regression approach of Zou [29] with both unadjusted and adjusted analyses (adjusted for gender, depression, mean-centred age, and SOC-29). Results are presented separately for 3 and 6 years as risk ratio and 95% CI. The ANCA and reference cohort were compared with regard to the prevalence of impaired QoL by means of odds ratios, 95% confidence intervals, and *p*-values. The association between QoL and the number of dysfunctions (bowel, urinary and sexual) that gives rise to major bother (none, one, two, or three) was assessed using a logistic regression model with number, subgroup (3- and 6-year follow-up and reference population) and number subgroup as fixed effects and the same variables for adjustment as previously. Results were presented graphically by the estimated risks of impaired QoL and 95% CI. Parameter estimation was done using the Glimmix and Genmod procedures in SAS version 9. (SAS Institute Inc., Cary, NC, USA) and graphics with ggplot in R version 3.6.3 [25].

## Results

**Fig. 1:**
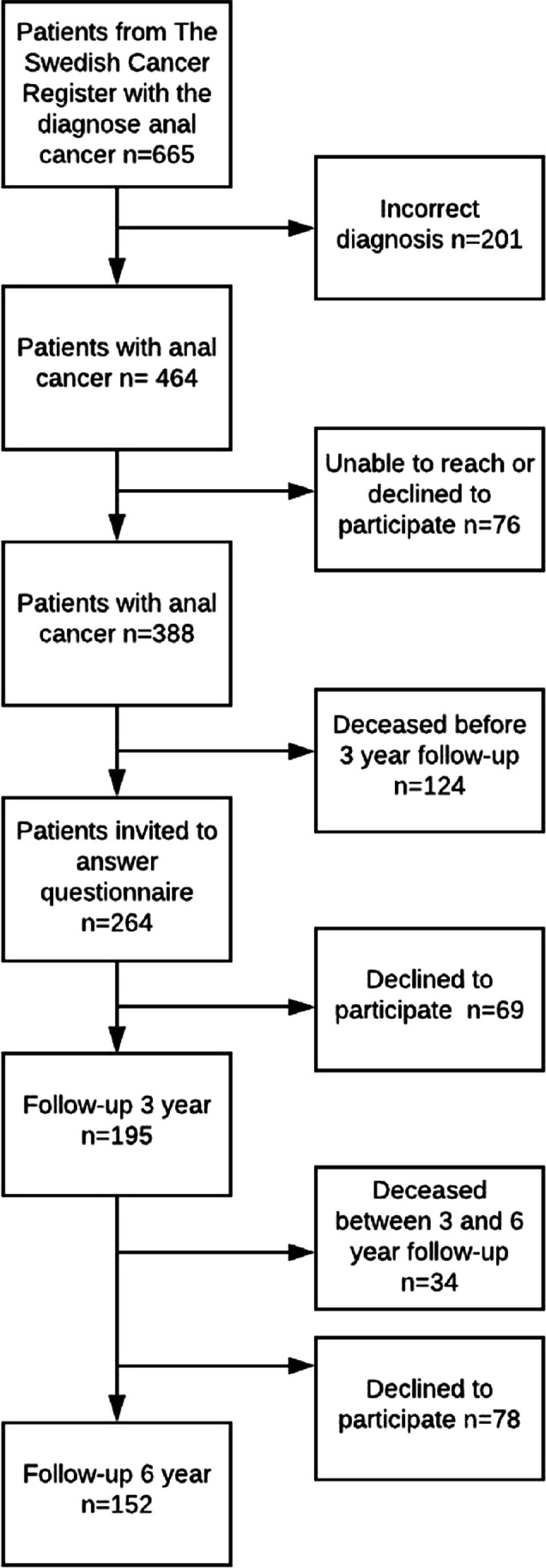
In total, 195 responded to the 3-year follow-up questionnaire and 152 to the 6-year follow-up questionnaire. Nine patients who declined participation at 3 years participated and responded at 6 years participated and responded at 6 years. In total, 204 unique patients responded to at least one questionnaire

From an original cohort of 464 patients with anal cancer, 264 were alive and contacted at 3 years and 230 at 6 years. In total 195 patients (74%) responded to the 3-year questionnaire and 152 (66%) to the 6-year questionnaire (Fig. 1). Nine patients who declined participation at 3 years participated and responded at 6 years. In total, 204 unique patients responded to at least one questionnaire. In Table 1 demography, clinicopathological and treatment details are presented. More than 60% were current or previous smokers. Forty-three percent of patients had tumors with nodal involvement at diagnosis. The majority of patients received chemoradiotherapy with curative intention (Table 1).

**Table 1:** Demography

Variable	Questionnaire responders* (*n* = 204)
Age (median)	64
Gender	
Male	46 (23%)
Female	158 (78%)
Marital status	
In a relationship	175 (86%)
Not in a relationship	20 (10%)
Missing	9 (4%)
Occupation	
Working	59 (29%)
Retired	132 (65%)
Unemployed or student	6 (3%)
Sick leave (full or part time)	24 (12%)
Smoking	
Current smoker	31 (15%)
Previous smoker	95 (47%)
Never smoker	74 (36%)
Missing	4 (2%)
Comorbidity	
Diabetes	14 (7%)
Hypertension	58 (28%)
Cardiovascular disease	26 (13%)
Cerebrovascular disease	4 (2%)
Renal dysfunction	2 (1%)
COPD/asthma	11 (5%)
HIV-positive	2 (1%)
Immunosuppression	9 (4%)
Stoma	
Yes	49 (24%)
No	146 (72%)
Missing	9 (4%)
TNM staging (AJCC 7th edition)	
0	6 (3%)
I	31 (15%)
II	76 (37%)
III A	31 (15%)
III B	52 (26%)
IV	3 (2%)
Missing	5 (3%)
Initial treatment strategy^1^	
Curative	201 (99%)
Palliative	0 (0%)
Missing	3 (2%)
Type of primary treatment^2^	
Chemoradiotherapy	102 (50%)
Radiotherapy	48 (24%)
Chemotherapy followed by radiotherapy	39 (19%)
Chemotherapy	1 (1%)
Surgery	7 (3%)
Surgery + adjuvant treatment	2 (1%)
Best supportive care	5 (2%)
Type of chemotherapy	(*n* = 144)
Mitomycin + 5FU	100 (69%)
Platinum + 5FU	40 (28%)
Other	4 (3%)
Radiotherapy total gray	(*n* = 192)
< 55 Gray	37 (19%)
55–60 Gray	82 (43%)
> 60 Gray	73 (38%)

^1^Decision from multidisciplinary conference

^2^Treatment received

*Participants who have responded to at least one questionnaire at 3- or 6-year follow-up. (Three years n = 195, 6 years n = 152, 9 participants responded only at 6-year questionnaire)

**Fig. 2:**
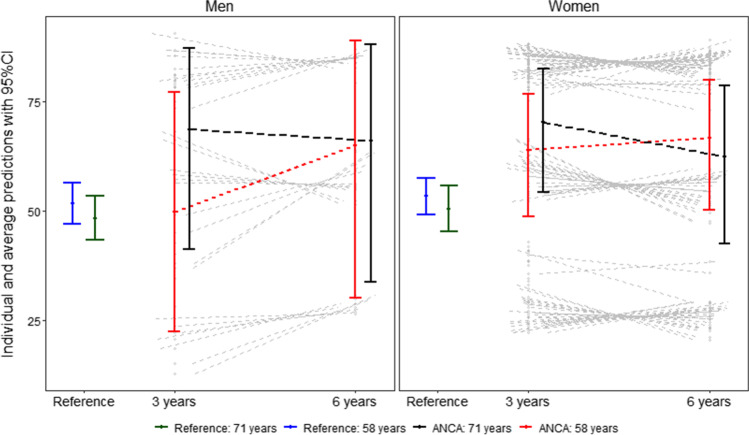
QoL Adjusted mean predictions for the risk of low QoL at 3 and 6 years in the ANCA cohort and in the reference population (“Reference”). The adjustment variable age is set at first and third quantiles of age at inclusion, 58 and 71 years, respectively. The figure demonstrates the individual and average risk of impaired quality of life with 95% confidence intervals. As benchmark, the corresponding risk in the reference group is displayed with 95% CI

Overall, 60% of patients with anal cancer reported low QoL at both 3 and 6 years with no tendency to change over time. In the reference population, low QoL was reported in 52% (Fig. 2), and this did not differ from the patient population. In Table 1, the potential explanatory variables sense of coherence, depression, and level of bother from functional impairment are presented. Both senses of coherence and depression appeared stable over time. The rate of depression was slightly higher among patients compared to the reference population, at 3 years with 19% and at 6 years with 17%, compared to controls with 14%. The reference population reported major bother from bowel (13%), urinary (13%), and sexual function (18%). The patients reported a higher degree of bother regarding all functions (bowel 51%, urinary 33%, and sexual function 26%). Although no statistical comparisons were made, it seems as if bother of urinary function remained high over time, whereas bother regarding anal pain, sexual function and bowel function seemed to decrease somewhat over time (Table 2).

**Fig. 3:**
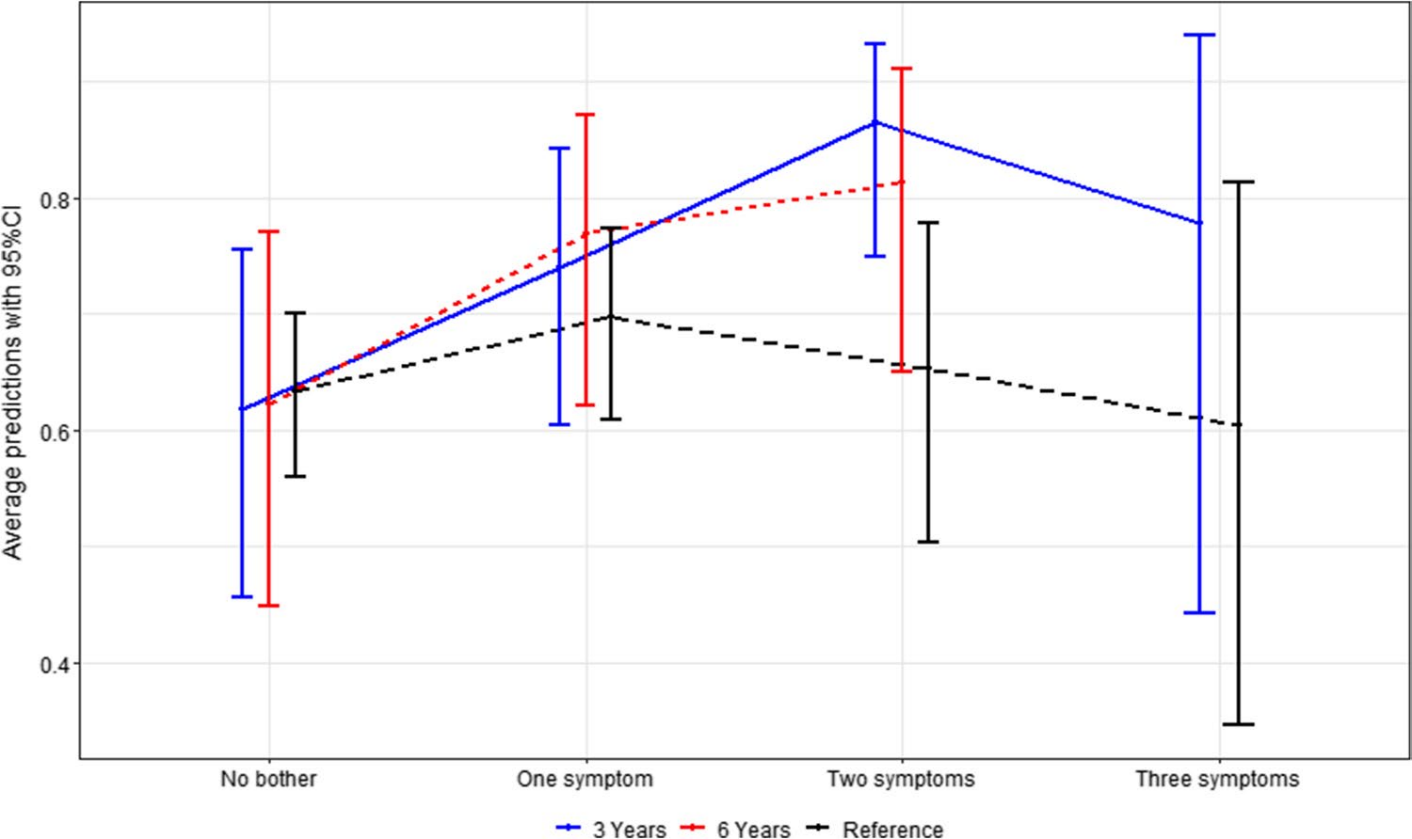
Correlation between numbers of bothers There are no patients reporting 3 bothers of symptoms at the 6-year follow-up

Level of bother as an explanatory variable for low QoL was explored with regression analysis presented in Table 3. Impaired QoL was more prevalent among patients with major bother due to bowel dysfunction (at 3 years RR 1.42, 95% CI (1.06–1.9) *p*-value 0.020, at 6 years RR 1.52, 95% CI (1.03–2.24) *p*-value 0.034) and urinary dysfunction (at 6 years RR 1.44, 95% CI (1.08–1.91) *p*-value 0.013). The correlation between numbers of bothers regarding impaired functions, and QoL is reported in Fig. 3. In the reference population, no obvious relationship between numbers of bother responses, and QoL was observed; whereas for patients, there was a tendency for a positive relationship between numbers of bodily functions causing bother and risk of impaired QoL for those reporting one and two bothers of functions. We only found patients reporting three bothers of function at 3 years and not at 6 years and with a decreasing tendency of relationship with QoL.

**Table 2:** Outcome variables—QoL, sense of coherence, depression, and bother

Variable	Category	Reference (*n* = 1078)	(%)	3 years (*n* = 195)	(%)	6 Years (*n* = 152)	(%)
QoL	Good	511/1062	48	76/188	40	59/146	40
	Low	551/1062	52	112/188	60	87/146	60
	Missing	16/1078	2	7/195	4	6/152	4
Sense of Coherence	Median (Q1;Q3)	154 (138;167)		155 (141;167)		155 (138.5;169)	
Depressed	No	919/1068	86	157/194	81	126/151	83
	Yes/don’t know	149/1068	14	37/194	19	25/151	17
	Missing	10/1078	1	1/195	1	1/152	1
	n	1066		190		148	
Bother of bowel function	No bother	717/1060	77	42/186	23	39/148	26
	Minor bother	202/1060	19	49/186	26	44/148	30
	Major bother	141/1060	13	95/186	51	65/148	44
	Missing	18/1078	2	9/195	5	4/152	3
Bother of urinary function	No bother	735/1057	70	84/193	44	50/149	34
	Minor bother	188/1057	18	46/193	24	52/149	35
	Major bother	134/1057	13	63/193	33	47/149	32
	Missing	21/1078	2	2/195	1	3/152	2
Bother of sexual function	No bother	658/1038	63	121/187	65	107/148	72
	Minor bother	195/1038	19	18/187	10	15/148	10
	Major bother	185/1038	18	48/187	26	26/148	18
	Missing	40/1078	4	8/195	4	4/152	3
Bother of anal pain	No bother			125/189	66	105/151	70
	Minor bother			28/189	15	27/151	18
	Major bother			36/189	19	19/151	13
	Missing	1078/1078	100	6/195	3	1/152	1

**Table 3:** Regression analysis

Variable	Year	Low QoL in each bother categories							Unadjusted		Adjusted	
		Patients with low QoL/no bother of function	Patients with low QoL/minor bother of function	Patients with low QoL/major bother of function	Missing				RR (95% CI)	p-value	RR (95% CI)	p-value
Bother of bowel function	3	18/41 (44%)	22/47 (47%)	67/93 (72%)	14 (7%)	Major bother	vs	No bother	1.64 (1.14–2.37)	0.008	1.54 (1.06–2.23)	0.023
						Major bother	vs	Minor bother	1.54 (1.11–2.14)	0.010	1.42 (1.06–1.9)	0.020
						No bother	vs	Minor bother	0.94 (0.59–1.49)	0.785	0.92 (0.6–1.42)	0.713
	6	23/36 (64%)	18/42 (43%)	44/65 (68%)	9 (6%)	Major bother	vs	No bother	1.06 (0.79–1.43)	0.703	1.01 (0.75–1.36)	0.932
						Major bother	vs	Minor bother	1.58 (1.07–2.33)	0.021	1.52 (1.03–2.24)	0.034
						No bother	vs	Minor bother	1.49 (0.97–2.28)	0.067	1.5 (1–2.25)	0.048
Bother of urinary function	3	41/83 (49%)	28/44 (64%)	41/59 (69%)	9 (5%)	Major bother	vs	No bother	1.41 (1.07–1.85)	0.015	1.24 (0.96–1.61)	0.095
						Major bother	vs	Minor bother	1.09 (0.83–1.45)	0.538	1.01 (0.77–1.32)	0.949
						No bother	vs	Minor bother	0.78 (0.57–1.06)	0.112	0.81 (:0.6–1.09)	0.166
	6	23/48 (48%)	26/51 (51%)	36/45 (80%)	8 (5%)	Major bother	vs	No bother	1.67 (1.2–2.32)	0.002	1.56 (1.13–2.15)	0.007
						Major bother	vs	Minor bother	1.57 (1.16–2.13)	0.004	1.44 (1.08–1.91)	0.013
						No bother	vs	Minor bother	0.94 (0.63–1.4)	0.761	0.92 (0.65–1.32)	0.653
Bother of sexual function	3	66/114 (58%)	11/18 (61%)	30/48 (63%)	15 (8%)	Major bother	vs	No bother	1.08 (0.82–1.41)	0.578	1.22 (0.92–1.62)	0.173
						Major bother	vs	Minor bother	1.02 (0.67–1.57)	0.918	0.96 (0.64–1.44)	0.846
						No bother	vs	Minor bother	0.95 (0.63–1.41)	0.791	0.79 (0.54–1.14)	0.205
	6	62/102 (61%)	6/14 (43%)	17/26 (65%)	10 (7%)	Major bother	vs	No bother	1.08 (0.78–1.48)	0.655	1.14 (0.81–1.61)	0.459
						Major bother	vs	Minor bother	1.53 (0.78–2.97)	0.214	1.41 (0.75–2.66)	0.293
						No bother	vs	Minor bother	1.42 (0.76–2.65)	0.273	1.23 (0.68–2.22)	0.485
Bother of anal pain	3	63/121 (52%)	20/27 (74%)	26/36 (72%)	11(6%)	Major bother	vs	No bother	1.39 (1.06–1.81)	0.016	1.36 (1.06–1.73)	0.015
						Major bother	vs	Minor bother	0.98 (0.72–1.32)	0.869	1.04 (0.78–1.41)	0.775
						No bother	vs	Minor bother	0.7 (0.53–0.93)	0.014	0.77 (0.58–1.02)	0.065
	6	55/101 (54%)	18/26 (69%)	14/19 (74%)	6 (4%)	Major bother	vs	No bother	1.35 (0.98–1.87)	0.066	1.33 (0.92–1.93)	0.130
						Major bother	vs	Minor bother	1.06 (0.73–1.54)	0.742	1.12 (0.75–1.68)	0.581
						No bother	vs	Minor bother	0.79 (0.58–1.07)	0.132	0.84 (0.62–1.14)	0.265

(3-year population: *n* = 195, 6-year population: *n* = 152)

## Discussion

Radiotherapy causes a continuous tissue damage that might increase over time and negatively affect body functions. Our hypothesis was that QoL would deteriorate between 3 and 6 years after diagnosis in patients treated for anal cancer. Although results from the investigated cohort may indicate a lower QoL following treatment for anal cancer compared to a reference population, deterioration over time could not be confirmed.

However, patients experiencing bother from functional symptoms had lower QoL, and this correlation was more apparent for bowel and urinary function. Our findings were corroborated by those found in a Danish study where 21% of patients reported great distress due to urinary dysfunction [24]. Additionally, we found indications that patients who experienced major bother with bowel function had a reduced QoL both at 3 and 6 years consistent over time. Bother with bowel function has been reported before in patients with anal cancer [19, 22, 24], but it is important to stress that there was no clear improvement over time. Our findings that anal pain had a tendency to cause major bother as well as low QoL, indicating that it is important to address this at follow-up.

We did not find any difference in QoL related to level of bother with sexual function. Previous studies have reported impaired sexual function as one of the most common problems, but perhaps bother of sexual function is not as important for overall QoL as previously thought [19]?.

The results of the study confirmed our second hypothesis, that low QoL is correlated to one or more functional symptoms causing bother. This finding has not previously been reported in relation to patients with anal cancer. We also found that bother of some functions seemed to be more important than others. Similar research has been performed in the prostate cancer field with comparable results, and in one study bother relating to bowel and urinary function tended to become worse over time and in turn negatively affected QoL, while bother regarding sexual dysfunction was not perceived as equally severe, nor did it affect the QoL to the same extent [9]. It is possible that this is due to the imperative nature of a good bowel and urinary function. It might be that disturbed bowel and urinary function impacts on social interaction and that this in turn causes bother, while the loss of ability to have sex, even though of great importance to many, may be more easily accepted.

This study is unique in the context of anal cancer as it relates to the bother patients perceive rather than the actual functional symptoms, giving the patients the prerogative to decide what they find important. One possible reason for no clear deterioration of QoL over time might be that the patients adjust over time to their functional symptoms resulting in less bother and no deterioration. This phenomenon is described in a conceptual framework called response shift theory [20]. Response shift is thought of as a positive adaptive process occurring over time. Patients report better outcomes over time, not because they are “objectively” doing better, but because they have adapted psychologically and match their new life circumstances in order to better cope with them. Response shifting is considered to involve a re-prioritization of values. The effects of this process could be seen as a potential source of bias, if one wished to quantify the long-term side effects of a given treatment. In QoL research, however, response shifting is considered to be fundamental to understand patient-reported and perceived QoL [11, 12]. Taken together with the fact that bother remained over time, as did reduce QoL, it could be of value to offer follow-up after more than 3 years for patients with anal cancer.

Strengths with this study include the nationwide cohort, the relatively large number of patients included, the high response rate, and the longitudinal design with considerably longer follow-up than usually reported in studies on anal cancer survivorship. Another strength with in this study is the use of an anal cancer-specific questionnaire compared with earlier studies using more generic instruments. In further research, it would also be of great interest to use the EORTC QLQ-ANL27 [19].

One limitation is the lack of a baseline questionnaire, which would have facilitated detection of patient-related factors influencing QoL prior to treatment. Another limitation is that there are some differences between the ANCA cohort and the reference population, even though we have corrected for age there is a possibility that the older age in the ANCA cohort that renders more patients to be retired my affect overall QoL when comparing groups. It must also be considered that the patients that did not respond to the questionnaire but that were still alive were somewhat older and more co-morbid, which must be taken into account when extrapolating results.

Another limitation is the diverse treatment schedules applied, due to a lack of national guidelines and various treatment traditions at different centers in Sweden during the study period. A national treatment guideline, a national multidisciplinary conference, and centralization to four university hospitals were introduced in 2017. Whether this will have an impact on QoL remains to be studied.

## Conclusions

Bother regarding bodily functions are of importance to QoL, and our study indicates that clinical follow-up should include routine questions on function as well as self-perceived bother in order to identify and treat symptoms and dysfunctions and possibly thereby improve QoL.

## Data Availability

Data is stored by authors and may be available upon request.

## Code availability

N/A.
